# 3D printed scaffold for repairing bone defects in apical periodontitis

**DOI:** 10.1186/s12903-022-02362-4

**Published:** 2022-08-08

**Authors:** Cong Li, Xiaoyin Xu, Jing Gao, Xiaoyan Zhang, Yao Chen, Ruixin Li, Jing Shen

**Affiliations:** 1 Tianjin Key Laboratory of Oral and Maxillofacial Function Reconstruction, Tianjin Stomatological Hospital, The Affiliated Stomatological Hospital of Nankai University, No.75, Dagu Road, Heping District, Tianjin, 300041 China; 2 The Affiliated Stomatological Hospital of Soochow University, Suzhou, 215000 Jiangsu Province China

**Keywords:** PLGA sustainably released microspheres, Low-temperature deposition 3D printing technology, Periapical periodontitis, Bone defect, New bone regeneration

## Abstract

**Objectives:**

To investigate the feasibility of the 3D printed scaffold for periapical bone defects.

**Methods:**

In this study, antimicrobial peptide KSL-W-loaded PLGA sustainable-release microspheres (KSL-W@PLGA) were firstly prepared followed by assessing the drug release behavior and bacteriostatic ability against *Enterococcus faecalis* and *Porphyromonas gingivalis*. After that, we demonstrated that KSL-W@PLGA/collagen (COL)/silk fibroin (SF)/nano-hydroxyapatite (nHA) (COL/SF/nHA) scaffold via 3D-printing technique exhibited significantly good biocompatibility and osteoconductive property. The scaffold was characterized as to pore size, porosity, water absorption expansion rate and mechanical properties. Moreover, MC3T3-E1 cells were seeded into sterile scaffold materials and investigated by CCK-8, SEM and HE staining. In the animal experiment section, we constructed bone defect models of the mandible and evaluated its effect on bone formation. The Japanese white rabbits were killed at 1 and 2 months after surgery, the cone beam computerized tomography (CBCT) and micro-CT scanning, as well as HE and Masson staining analysis were performed on the samples of the operation area, respectively. Data analysis was done using ANOVA and LSD tests. (α = 0.05).

**Results:**

We observed that the KSL-W@PLGA sustainable-release microspheres prepared in the experiment were uniform in morphology and could gradually release the antimicrobial peptide (KSL-W), which had a long-term antibacterial effect for at least up to 10 days. HE staining and SEM showed that the scaffold had good biocompatibility, which was conducive to the adhesion and proliferation of MC3T3-E1 cells. The porosity and water absorption of the scaffold were (81.96 ± 1.83)% and (458.29 ± 29.79)%, respectively. Histological and radiographic studies showed that the bone healing efficacy of the scaffold was satisfactory.

**Conclusions:**

The KSL-W@PLGA/COL/SF/nHA scaffold possessed good biocompatibility and bone repairing ability, and had potential applications in repairing infected bone defects.

*Clinical significance* The 3D printed scaffold not only has an antibacterial effect, but can also promote bone tissue formation, which provides an alternative therapy option in apical periodontitis.

**Supplementary Information:**

The online version contains supplementary material available at 10.1186/s12903-022-02362-4.

## Introduction

The periapical disease is a common oral complaint with high prevalence in stomatology which can not only cause toothache and chewing discomfort, but also lead to the destruction of the apical alveolar bone. If not treated promptly, it may eventually lead to the extraction of the affected teeth and even disturb the adjacent teeth. The vast majority of periapical disease is mainly caused by the infection of a variety of bacteria in the root canal spreading to the periapical tissues, leading to the prolonged inflammation, and the destruction of the surrounding bone. Although most of the periapical lesions of the affected teeth can be cured by root canal treatment (RCT), there are still some teeth whose alveolar bone destruction has not stopped. In addition to nonsurgical endodontic retreatment, endodontic microsurgery (EMS) is an alternative approach to preserve the tooth and avoid the extraction. EMS is a treatment method characterized by modern microsurgery technology, which combines the use of an operating microscope or an endoscope, the preparation of the root-end cavity of the ultrasound tip, and a more biocompatible root end filling material, such as mineral trioxide aggregate (MTA), bioceramic and so on. When the area of bone defect caused by periapical inflammation is too large, the treatment of bone substitute materials is very necessary to obtain a relatively good prognosis, which helps to accelerate the healing process of the lesion. Currently, the material for repairing bone tissue is an important subject which interests the scholars at home and abroad. A variety of bone substitution materials have been developed for the repair of maxillofacial bone defects, such as hydroxyapatite, titanium, tissue-engineering bone scaffold and so on. However, it is a pity that by now there is no bone graft materials can fully meet the requirements of bone repair materials. For example, Bio-Oss® particles, which is currently the most widely used, has no active components such as osteogenic factors, so it lacks bone generation and bone inductivity, also, the natural absorption in vivo degradation is a long process [1]. Therefore, it is of great significance to develop biomedical materials with satisfactory antimicrobial and osteogenic activities.

However, the research and development of materials with suitable three-dimensional structure, proper degradation rate and may promote bone regeneration has always been the focus and difficulty of research. At present, it is feasible to construct the bone tissue engineering scaffolds with the same morphology as the tissue defect area. In our previous studies, the 3D printed scaffold containing collagen, fibroin and nano-hydroxyapatiteand has excellent biocompatibility and osteogenic activity. From the perspectives of material science, collagen as the organic component of natural bone matrix, and silk fibroin as the product extracted from silk, both have extensive sources and good bio-compatibility [2, 3]. Hydroxyapatite, as one of the components of natural bone matrix, has certain osteoconductivity, but it also has disadvantages such as low compressive strength and poor inducing activity [4]. Therefore, we mix the collagen/silk fibroin/nano-hydroxyapatite together in order to reinforce complementary advantages.

Periapical periodontitis is an inflammatory disease in which the presence of bacteria not only increases the risk of infection but also affects the healing of bone defects. Therefore, inhibition of the bacterial growth and propagation is of great significance when it comes to promoting the healing effect of bone substitutes in cases of high infection risk. At present, post operative antibiotics are commonly prescribed to prevent wound or graft infections, but there are risks of side effects and possible antibiotic-resistance. Previously, metal ions have been introduced into scaffold materials to prevent bone tissue infection, but their potential tissue toxicity has limited their clinical application.

With more and more in-depth studies on antimicrobial peptides, it has been found that they have a certain killing effect on bacteria, fungi and viruses, but not easy to produce drug resistance. Among the synthetic antimicrobial peptides, KSL-W(KKVVFWVKFK-NH2) has the characteristics of lower poisonous and side effects, strong antiseptic qualities and no drug resistance during killing bacterial owing to its unique amino acid sequence [5–7], which makes it attractive candidate for drug development.

However, the direct addition of KSL-W to bone scaffolds can lead to drug burst release and cannot give play to long-term antibacterial effects. With the improvement of medical level, the sustained and controlled release delivery system has gradually entered the scene, the medicine in the pharmaceutical carriers can slowly emanate. At present, microspheres and microcapsules have been extensively studied in tissue engineering and pharmaceutical technology, which can be entrapped drugs wherein and prolong the time of drug action [8]. Poly (lactic co-glycolic acid) (PLGA) is a macromolecule polymer material with good biocompatibility and ball-forming performance [9, 10]. Since it has been approved by the FDA to be used on humans, it is widely used in the medicinal field, usually used as a scaffold for tissue engineering or as a carrier for various small molecule drugs, proteins and genes [11, 12]. With the gradual degradation of PLGA, the payloads are released and elicit a variety of biological functions in vitro and in vivo [13, 14].

**Fig. 1:**
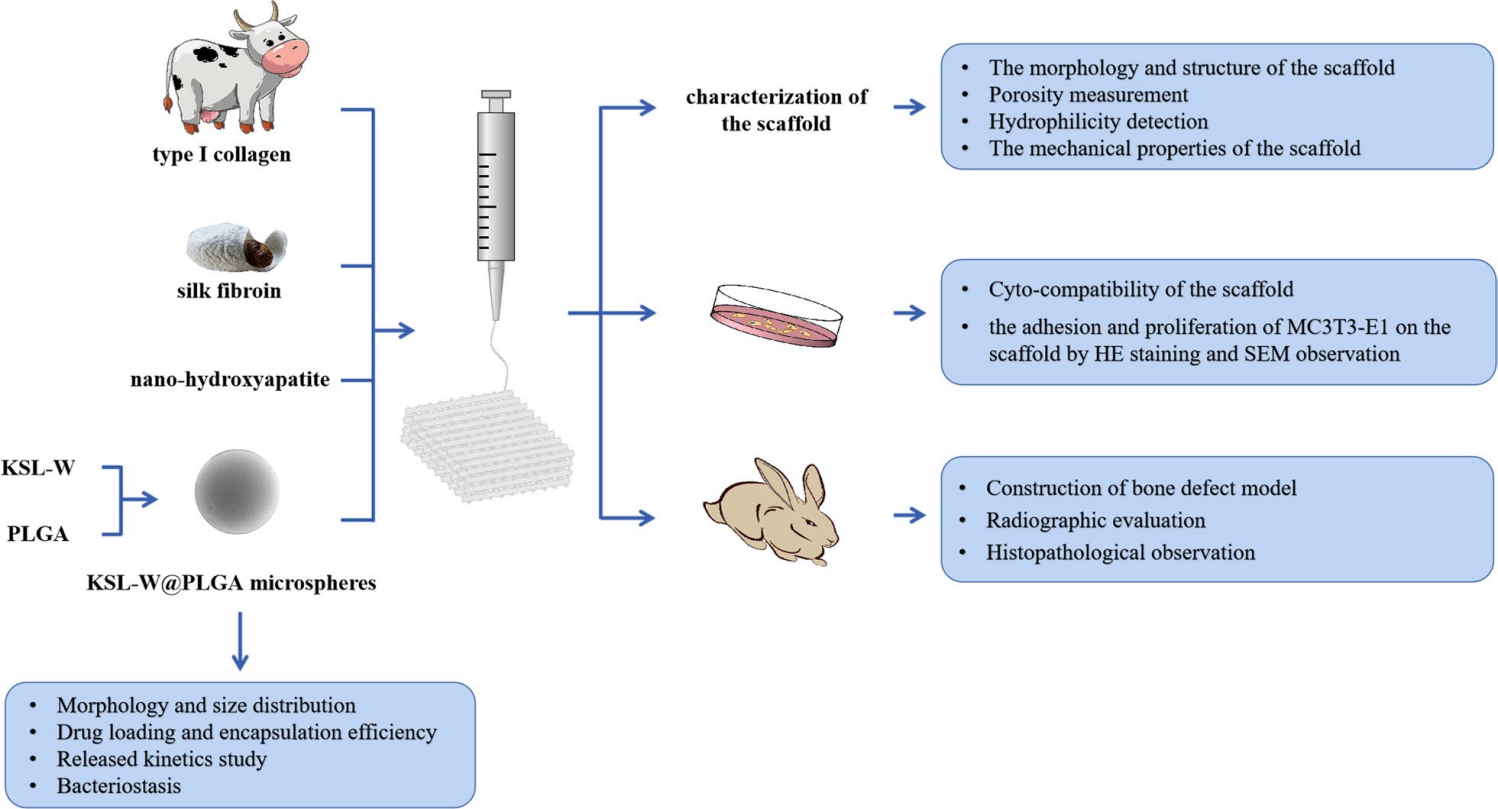
Schematics of the whole study

Therefore, this research intends to establish a drug delivery system (KSL-W@PLGA) with long-term bacteriostatic function, which can be combined with the previous 3D printed bone scaffold materials to achieve osteoconductive function and inhibit the growth of microorganisms simultaneously. Schematics of the whole study were shown in Fig. 1. This investigation is of great significance as it validates the antibacterial and osteogenic function of dual functional bone scaffolds in the infected bone defect model.

## Materials and methods

### Preparation and properties of KSL-W@PLGA microspheres

#### The proper preparation conditions of KSL-W@PLGA microspheres were selected by orthogonal test

##### The drafting of KSL-W calibration curve [15]

The 5 mg of KSL-W was weighed accurately and dissolved in 5 mL of ultra-pure water. After confirming that the maximum absorption wavelength of KSL-W is 225 nm with an ultraviolet (UV) spectrophotometer, KSL-W with concentration of 0.5 mg/mL, 0.25 mg/mL, 0.2 mg/mL, 0.1 mg/mL and 0.05 mg/mL was obtained by gradient dilution. Prepared standard solution above for calibration curve, and run UV–VIS program. Then draw a diagram with absorption and concentration.

##### Preparation of KSL-W@PLGA microspheres

**Fig. 2:**
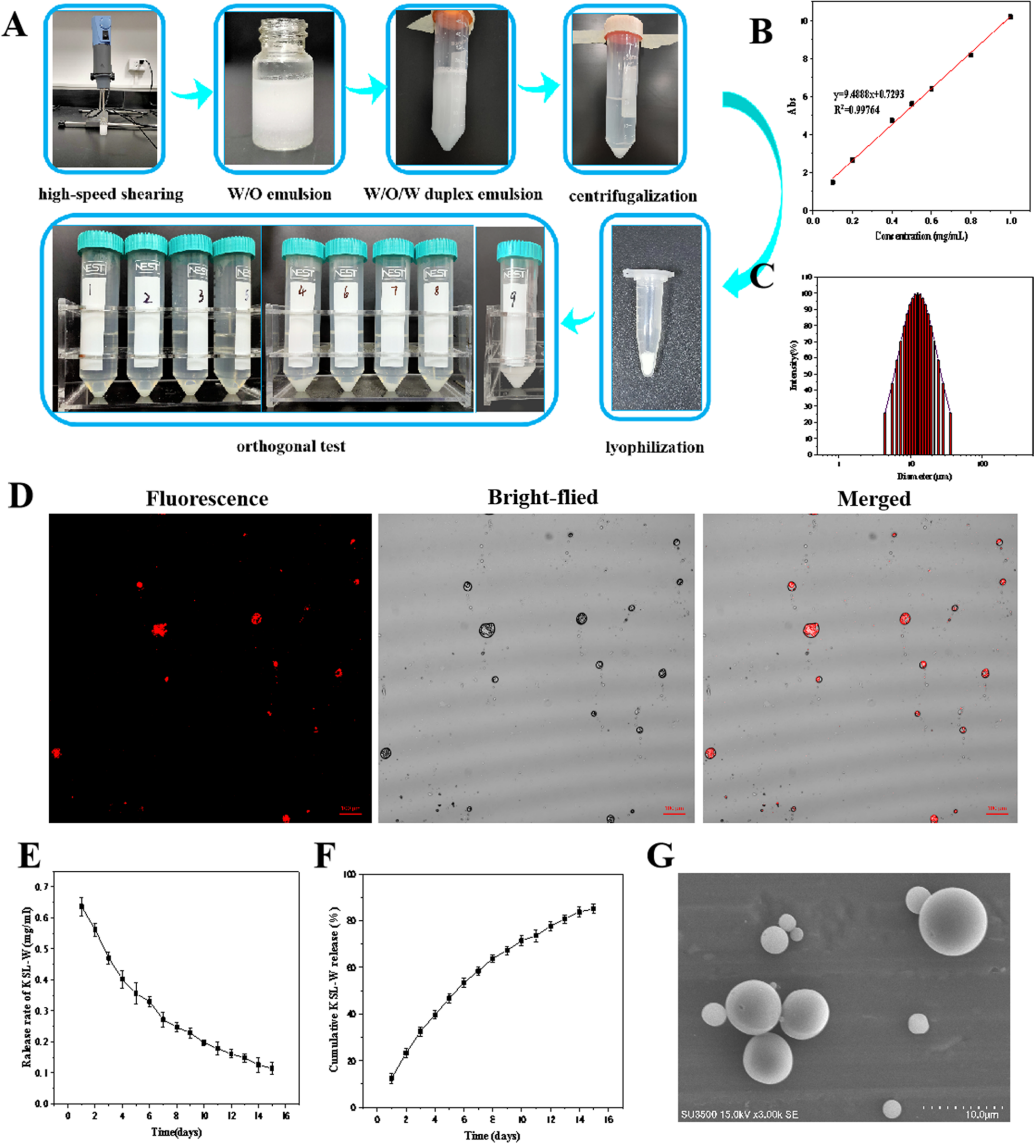
**A** The preparation process of KSL-W@PLGA microspheres; **B** Standard curve of KSL-W; **C** Particle size distribution of KSL-W@PLGA microspheres; **D** Distribution of KSL-W in PLGA microspheres under CLSM; **E** The sustained release concentration of KSL-W@PLGA microspheres; **F** The release curve of KSL-W@PLGA microspheres; **G** Surface morphology of KSL-W@PLGA microspheres under SEM

The microspheres were prepared by W/O/W emulsifying-solvent evaporation technique [16, 17]. First, the aqueous solution of KSL-W was injected into the dichloromethane solution (DCM) of PLGA, and emulsified it with a high-speed shearing machine for 2 min (15,000 r/min) to obtain the W/O emulsion. After that, the emulsion was added to the PVA solution and emulsified at 10,000 r/min for 1 min to obtain the W/O/W duplex emulsion. The microsphere suspension of KSL-W@PLGA was obtained by using a magnetic agitator (300 rpm, 6 h) to remove the excess DCM in the W/O/W emulsion. The suspension was centrifuged at 8000 rpm for 10 min, and the precipitates was washed with ultra-pure water for 3 times, then the KSL-W@PLGA microspheres were obtained by lyophilization (Fig. 2A).

However, morphology and release properties of microspheres are fundamentally influenced by the process conditions, including inner aqueous phase, the oily phase, the outer aqueous phase, polymer concentration, emulsifier, stirring speed, temperature, etc [18–22]. The effects of the following four factors: (A) the concentration of PLGA, (B) the concentration of KSL-W, (C) the concentration of PVA emulsion and (D) the volume ratio of inner aqueous phase to oily phase (w/o ratio) on the encapsulation efficiency of KSL-W@PLGA microspheres were investigated. carried on the orthogonal experiment to optimized the technique design (Additional file 1: Table S1).

#### Morphology and size distribution of KSL-W@PLGA microspheres

Scanning electron microscope (SEM) was used to observe the surface morphology of KSL-W@PLGA microspheres [18]. The size distribution was determined with laser particle size analyzer. The distribution of KSL-W in PLGA microspheres was observed by confocal laser scanning microscope (CLSM), and the excitation light was set at 415 nm [18].

#### Drug loading and encapsulation efficiency of KSL-W@PLGA microspheres

The drug loading and encapsulation efficiency of the KSL-W@PLGA microspheres were calculated by using the UV spectrophotometer and the calibration curve of KSL-W measured above [23]. The specific operation method was as follows:

10 mg of KSL-W@PLGA microspheres were weighed and dissolved in 1 mL of DCM, which were completely dissolved in an ultrasound bath, and centrifuged at 10,000 rpm for 5 min. The absorbance of the supernatant was measured.

Drug loading (%) = the weight of KSL-W in microspheres/the weight of KSL-W@PLGA × 100%

Encapsulation efficiency (%) = the weight of KSL-W in microspheres/the weight of total KSL-W × 100%

#### Released kinetics study of controlled-release microspheres of KSL-W loading in vitro

Add 100 mg KSL-W@PLGA microspheres and 1 mL of 1 × PBS into a 5 mL centrifuge tube. Then the tube was placed on an incubator shakers at 37 °C, 150 rpm for 1d, 2d, 3d, 4d, 5d, 6d, 7d, 8d, 9d, 10d, 11d, 12d, 13d, 14d, 15d. After taking supernatant for each centrifugation, 1 mL of 1 × PBS was added to the centrifuge tube [22]. The concentration of KSL-W in supernatants was calculated according to the calibration curve of KSL-W.

#### Bacteriostasis of KSL-W@PLGA microspheres

The bacteriostatic activity of KSL-W@PLGA microspheres against *E. faecalis* and *Porphyromonas gingivalis* was evaluated by Oxford cup method [24].

The steps were as follows:

*Enterococcus faecalis* and *P. gingivalis* stored at − 80 °C were inoculated into Columbia Blood Agar and incubated under anaerobic conditions. *Enterococcus faecalis* was inoculated in BHI medium, *P. gingivalis* was inoculated in BHI medium containing hemin and vitamin K, and cultured at 37 °C for 16–18 h.

To analyse the antibacterial activity, after 200 μL of bacterial suspension was painted onto the Columbia Blood Agar, 200 μL sustained-release solution of KSL-W@PLGA microspheres on the 5th and 10th day was added into Oxford cup (8 × 6 × 10 mm), respectively. In addition, diameters of the bacteria inhibiting loop were recorded after 7 days of anaerobic incubation.

### The preparation and characterization of KSL-W@PLGA/COL/SF/nHA scaffold

#### Preparation of COL

The COL was fabricated according to our previously reported method [25–27]. Bovine tendon was purchased from a local slaughter house. Briefly, fat from the fresh bovine tendon was removed, and the residues were crushed and soaked in 0.05 mol/l Tris buffer for 24 h. The collected precipitate was added to acetic acid solution containing pepsin, and the collected supernatant was added to 3.5 mol/L NaCl solution for salting out. Then the resulting precipitate was collected, and exhaustively dialyzed for 5 days with deionized water at 4 °C.

The concentration of collagen solution was approximately 14.5 wt%, calculated as follows: (M_2_−M_0_)/(M_1_−M_0_) × 100%. A clean dry glass evaporating dish was taken and weighed as M_0_, and an appropriate amount of collagen solution was taken and weighed as M_1_. The evaporating dish containing collagen solution was put into a constant temperature drying oven at 60 °C for full drying, and weighed as M2. The above experiment was repeated 3 times and the average value was taken.

#### Preparation of SF

SF was extracted from silk according to a previously described method [25–27]. After boiling 100 g silk in 0.5% anhydrous Na2CO3 solution for 30 min for degumming, the silk was rinsed and dried in a constant-temperature dryer at 60 °C. The dried silk was put into a three-necked flask containing a 1:2:8 molar ratio of CaCl_2_ CH_3_CH_2_OH·H_2_O solution, and mechanically stirred for 2 h. After cooling, the mixed solution prepared above was dialyzed in running water, deionized water and 40% polyethylene glycol solution in sequence until the system was golden and translucent. After that, the solution was centrifuged at 8000 rpm for 15 min to remove the precipitate, and the supernatant was stored at 4 °C. The concentration of silk fibroin solution was about 3.3 wt% using the same method as the determination of collagen concentration.

#### Fabrication of KSL-W@PLGA/COL/SF/nHA scaffold

The scaffold was printed by importing a model of standard tessellation language (STL) format, which was designed using the design software Solidworks 2010, into the 3D printer [25–28]. The printing parameters were shown in the Additional file 1: Table S2.

The COL, SF, KSL-W@PLGA microspheres and nHA with a mass ratio of 4/5/2/1 were fully mixed, and centrifugation was performed for the printing of the scaffold. The printed and lyophilized scaffold was soaked in anhydrous ethanol for 24 h, followed by sodium hydroxide (pH = 10) for 24 h and ultra-pure water for 3 days. The scaffold after post-processing was flexible and elastic, and sterilized by exposure to 20 kGy Co.^60^

#### The character of KSL-W@PLGA/COL/SF/nHA scaffold

The morphology and structure of the scaffold were observed by SEM, while the modified liquid replacement method was used to calculate the porosity of the support samples. Porosity refers to the percentage of the volume of pores to the total volume of the material, which reflects the compactness of the material. The size of porosity not only affects the physical properties of materials, but also affects the transport of substances and the growth of cells [29]. The scaffold was placed in anhydrous ethanol with volume V_1_, and the volume V_2_ was recorded when no bubbles emerged from the scaffold after vacuum extraction. After the scaffold was removed, the volume V3 of the remaining anhydrous ethanol was recorded. The porosity of the scaffold was calculated according to the equation [25]:p (%) = (V1−V3)/(V2−V3) × 100%.

The water absorption of the scaffold was measured as follows [25]: the freeze-dried scaffold was weighed as M_1._ Surface moisture was blotted with filter paper after the scaffold was immersed in deionized water overnight, and the weight of this scaffold is W_2_. The calculation formula of water absorption is as follows: h (%) = (M_2_−M_1_)/M_1_ × 100%.

The tensile mechanical properties of the scaffold were measured using a universal testing machine (Instron5969, USA) and the compressive mechanical properties were measured using a universal testing machine (Instron5965, USA) [26]. The scaffold was cut into samples with a size of 30 mm × 10 mm × 2.7 mm, soaked in PBS solution for 24 h, and the tensile mechanical properties and compressive properties of materials were tested. The testing parameters of compression experiments were set as follows: The preload was 0.01 N, the speed was 5 mm/min, and the maximum compression strain was 30%. During tensile experiments, the tensile rate was set to 5 mm/min, while the stress–strain curve was displayed on the computer screen. Finally, the modulus of elasticity was calculated and the corresponding stress–strain curves were drawn out.

#### Cyto-compatibility of KSL-W@PLGA/COL/SF/nHA scaffold

The MC3T3-E1 cell was selected to study the cytotoxicity and biological compatibability of the scaffold [28]. The KSL-W@PLGA/COL/SF/nHA scaffold was gently placed on 24-well plates, and the MC3T3-E1 cell was seeded onto it at a density of 2 × 10^4^/mL and cultured for 3 days, 5 days, 7 days and 10 days respectively, and then 50 μL cck-8 solution was added to each well of the 24-well plate and incubated for 4 h, and measured spectrophotometrically at 450 nm wavelength.

#### HE staining and SEM observation on the adhesion and proliferation of MC3T3-E1 on the scaffold

The MC3T3-E1 cell was seeded onto the scaffold at a density of 2 × 10^4^/mL, after that, take out the scaffold on the 5th and 7th day of incubation. HE staining was performed after fixation with 4% paraformaldehyde solution, followed by paraffin embedding and serial sections at a thickness of 5 μm [27].

The scaffold was fixed with 2.5% glutaraldehyde fixative solution, followed by gradient dehydration, drying, conductive coating, and SEM was used to observe the cell morphology on the scaffold [27].

### Construction of mandibular bone defect model and evaluation the effect of bone formation

#### Construction of bone defect model

All experimental protocols were carried out in accordance with the procedures approved by the Animal Care and Use Committee of Tianjin Nankai Hospital. The Japanese white rabbit used in the experiment was purchased from Tianjin Yuda Experimental Animal Breeding Center Co., LTD..

Twenty-four skeletally mature female Japanese white rabbits (2.0–2.5 kg in weight) were used to establish an infected mandibular defect model representative of chronic periapical with bone destruction (n = 8 for each group) [28, 30]. The specific experimental groups were as follows (Additional file 1: Fig. S1):

The blank group: Bone defects were prepared without any material implanted.

The experimental group: The 5 mm × 5 mm × 2.7 mm scaffolds were placed in the operation area and covered with Bio-Gide® membrane.

The positive control group: The bone defects were filled with Bio-Oss® particles and covered with Bio-Gide® membrane.

In addition, three rabbits were sacrificed immediately after surgery and CBCT was taken to evaluate the consistency of the bone defect model (Additional file 1: Fig. S2).

#### Radiographic evaluation

CBCT and micro-CT images of rabbit mandible were taken at 4 and 8 weeks, respectively (n = 4 for each point). CBCT images were taken by 3DX550 Veraviewepocs, with a 360-degree rotation scanning. The tube voltage was 80 kV and the tube current was 5 mA. The exposure time was 9.4 s, the field of view was 80 mm × 80 mm, and three-dimensional images were obtained by image reconstruction when the layer thickness is 0.125 mm.

While, The scanning parameters of micro-CT were set as follows: the tube voltage was 50 kV, the core was 80 kV, and the maximum tube current was 0.5A, the tomography mode was set to the standard mode about 5.8 s/ rotation, and the spatial resolution was 7.5 μm@10%MTF, the reconstructed pixel size is 2 μm. Also, we used the corresponding software to analyze the morphometry data, including bone volume fraction (BV/TV), bone mineral density (BMD), trabecular number (Tb.N) and trabecular thickness (Tb.Th) obtained from region of interest (ROI).

#### Histopathological observation

On the fourth and eighth weeks of the experiment, the mandibles of the sacrificed rabbits were fixed in 4% paraformaldehyde buffer solution, then, these samples were soaked in paraffin after adequate decalcification, and cut using a microtome to obtain 5 μm thick sagittal sections. In this study, HE and Masson staining was used to evaluate the effects on the new bone formation.

#### Statistical analysis

SPSS software version 20.0 was used to perform the statistical analysis. Quantitative data were recorded as the mean ± standard deviation. Multiple comparisons were performed by combination of One-way analysis of variance (ANOVA) and the least significant difference (LSD) test. *p* < 0.05 represents a statistical difference.

## Results

### Preparation and properties of KSL-W@PLGA microspheres

#### The calibration curve of KSL-W

Since the maximum absorption wavelength of KSL-W is 225 nm, the UV spectrophotometer is used to measure the absorbance value of standard solutions at 225 nm, as shown in Additional file 1: Table S3.

According to the absorbance values in Additional file 1: Table S3, the standard curve and regression equation of KSL-W was protracted (Fig. 2B). The standard curve equation was y = 9.4888x + 0.7293, and the correlation coefficient or R value was 0.99.

#### The results of orthogonal test

The optimum technological conditions were determined by orthogonal testing and the range analysis method, the analysis of results was shown in Additional file 1: Table S4. The optimum preparation conditions of KSL-W@PLGA microspheres were obtained, which are as follows: the concentration of PLGA was 25 mg/mL, the concentration of KSL-W was 10 mg/mL, the concentration of PVA was 0.5%, w/o ratio was 2/1.

#### Morphology, size distribution, drug loading and encapsulation efficiency of KSL-W@PLGA microspheres

SEM showed that the microspheres prepared in the experiment had smooth surface and uniform size (Fig. 2C, G). Meanwhile, it was calculated that the drug loading and encapsulation efficiency of the microspheres were (5.29 ± 0.05)% and (74.23 ± 0.24)%, respectively.

As can be seen from Fig. 2G, the surface of KSL-W@PLGA microspheres prepared was smooth and smooth, but a few microspheres were still wrinkled, which may be caused by the powerful shearing force, but there was no adhesion between the microspheres. According to the observation results of CLSM, KSL-W could emit obvious fluorescence when the excitation light was 415 nm, which could intuitively evaluate the content of antimicrobial peptides wrapped in the microspheres. As shown in the Fig. 2D below, most of the prepared KSL-W@PLGA microspheres were coated with antimicrobial peptides, and only a few were empty PLGA microspheres.

#### Drug release of KSL-W@PLGA microspheres in vitro

The drug release amount and cumulative release amount at different time points are shown in Fig. 2E–F. As can be seen from Fig. 2E, the release rate in PBS buffer solution was increased significantly in the first 5 days, and then reached a flat, indicating that PLGA microspheres had a certain burst of release in the early stage. It could be seen from Fig. 2F that the drug release rate of the microspheres had reached 80% on the 15th day.

#### Bacteriostasis of KSL-W@PLGA microspheres

**Fig. 3:**
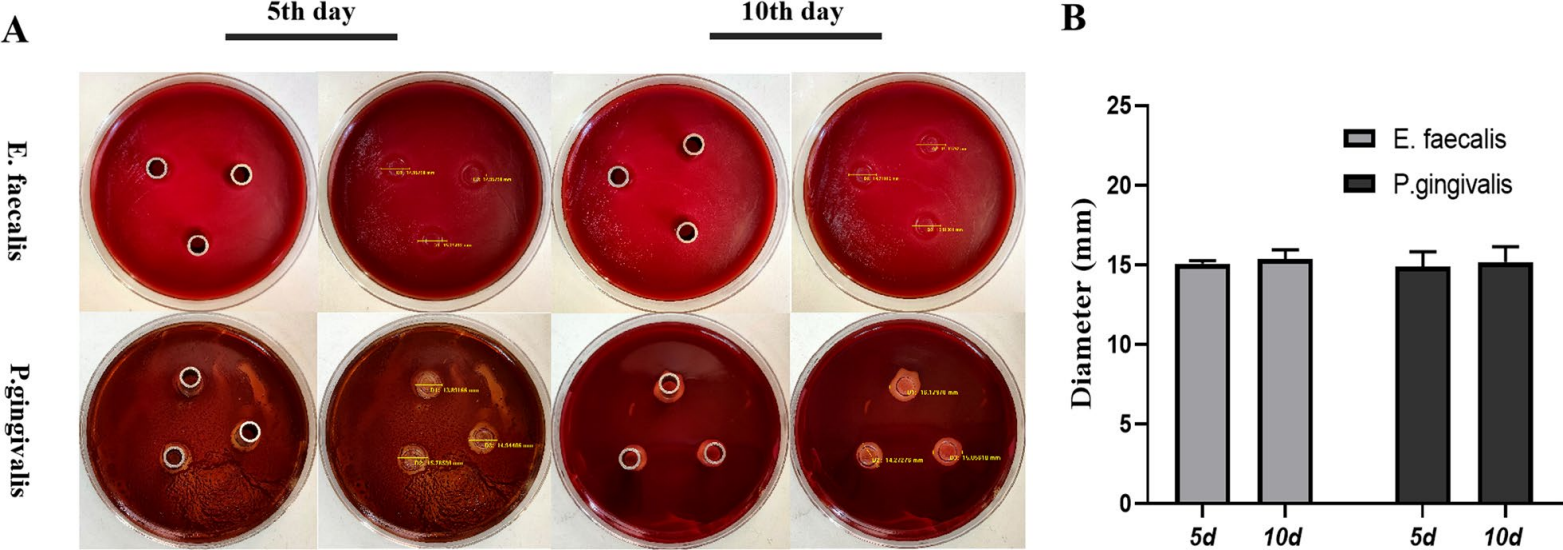
**A** Antibacterial effect of KSL-W@PLGA microspheres; **B** Statistical diagram of the antibacterial effect of KSL-W@PLGA microspheres

Oxford Cup method was used to determine the antibacterial activity of KSL-W@PLGA microspheres against the main pathogenic bacteria of chronic periapical periodontitis. The antibacterial effects against *E. faecalis* and *P.* *gingivalis* in vitro were shown in Fig. 3A.

The bacteriostatic efficacy can be seen in Fig. 3B below, sustained release solution of KSL-W@PLGA microspheres at both time points had significant bacteriostatic effects on *E. faecalis* and *P. gingivalis*, while, obvious bacterial inhibition appeared around Oxford cups. The inhibition zone diameter of sustained release solution on day 5 was 15.07 mm for *E. faecalis* and 14.87 mm for *P. gingivalis*, and on day 10 was 15.36 mm for *E. faecalis* and 15.16 mm for *P. gingivalis*.

### The preparation and characterization of KSL-W@PLGA/COL/SF/nHA scaffold

#### The character of KSL-W@PLGA/COL/SF/nHA scaffold

**Fig. 4:**
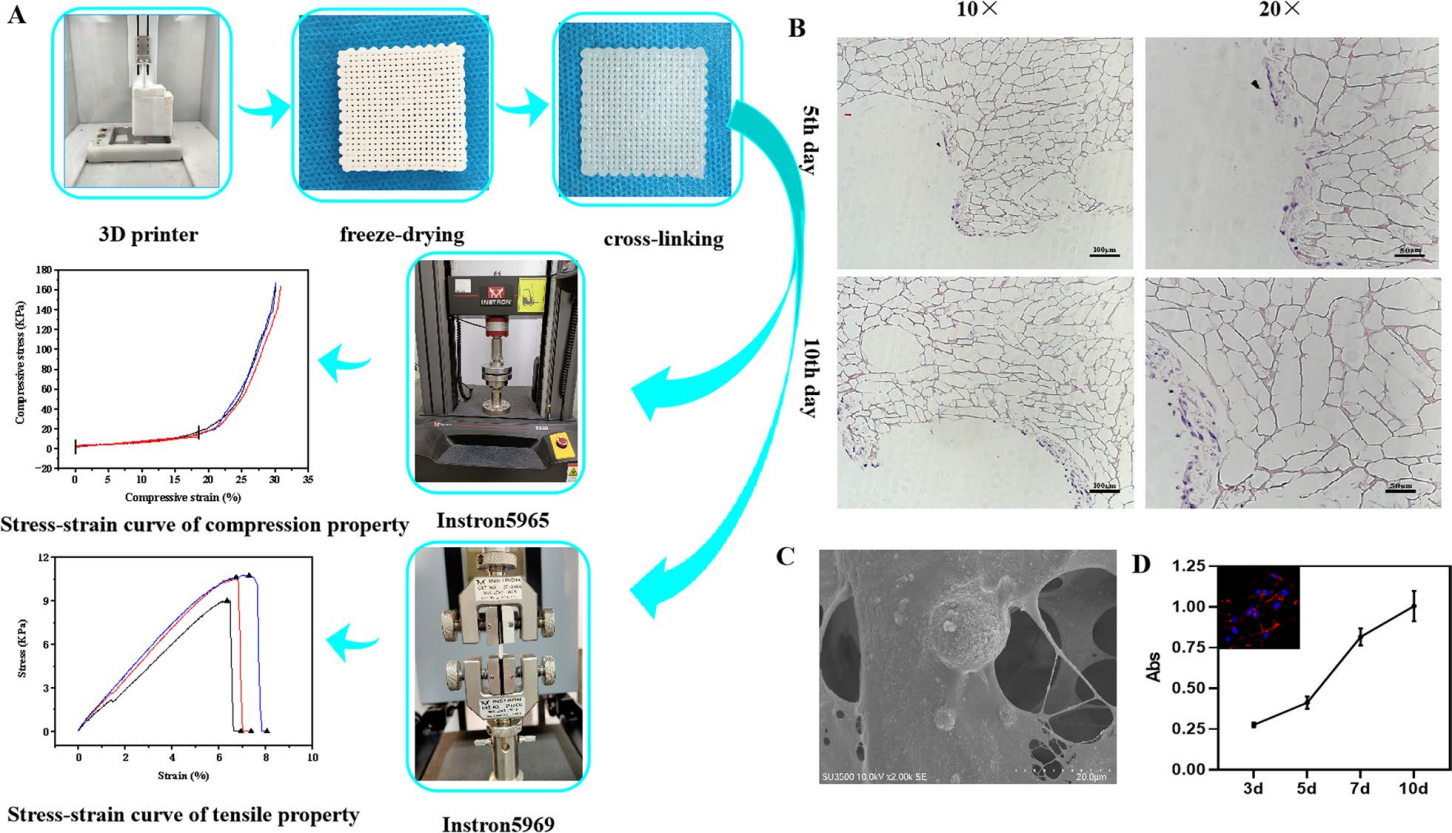
**A** Stress–strain curve of tensile property and compression property; **B** HE staining of MC3T3-E1 cells seeded onto the scaffold; **C** SEM image of the scaffold; **D** Growth curve of MC3T3-E1 on the scaffold (in the upper left corner is an CLSM image of MC3T3-E1 cells)

After lyophilization and post processing (Fig. 4A), the scaffold material was flexible and elastic. SEM showed that the 3D-printed scaffold had porous structure with good communication between pores, and the aperture was (523 ± 42) μm. Meanwhile, it could be observed that KSL-W@PLGA microspheres were scattered to the inside structure or on the surface of the scaffold (Fig. 4C). The porosity of the scaffold was (81.96 ± 1.83)%, and the water absorption was (458.29 ± 29.79)%.

As shown in Fig. 4A, the mechanical properties of the tensile test studied on a versatile testing machine showed that the elastic modulus of the 3D-printed scaffold materials was (155.38 ± 6.79) kPa. Also, the 3D-printed scaffold materials have a certain elasticity of compression, and its elastic modulus was (57.93 ± 4.32) kPa.

#### Cyto-compatibility of KSL-W@PLGA/COL/SF/nHA scaffold

After the MC3T3-E1 cells were inoculated on the scaffold with cell density of 2 × 10^4^/mL, we measured the absorbance values (A values) and drew the growth curve. It was found that the absorbance values increased with the extension of incubation time, and the cells proliferated most rapidly in the 5th-7th days, then slowed down (Fig. 4D).

#### HE staining and SEM observation on the adhesion and proliferation of MC3T3-E1 on the scaffold

HE staining was used to observe the growth of MC3T3-E1 cells. As can be seen from Fig. 4B, the cells showed a state of multilayer growth along the pores of the scaffold, and there was a significant increase of cells on the 7th day compared to the 5th day.

**Fig. 5:**
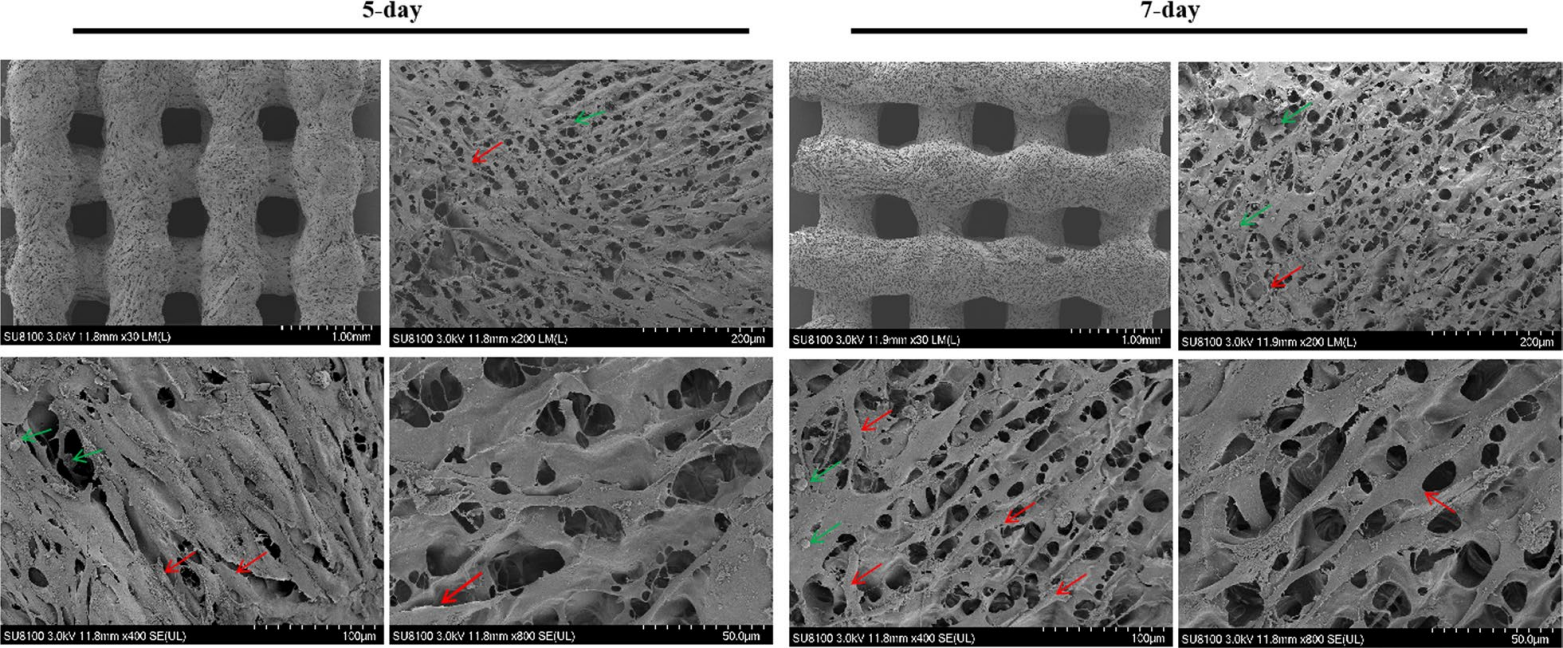
After 5-day and 7-day of mixed culture of the scaffold and MC3T3-E1 cells, the adhesion of MC3T3-E1 cells on the surface of the scaffold was observed by SEM. (Red arrow: MC3T3-E1 cells; Green arrow: KSL-W@PLGA microspheres)

SEM (Fig. 5) showed that MC3T3-E1 cells with good morphology adhered to the surface of the scaffold, the cells appeared polygonal shape and attached well with long processes.

### Construction of mandibular bone defect model and evaluation the effect of bone formation

**Fig. 6:**
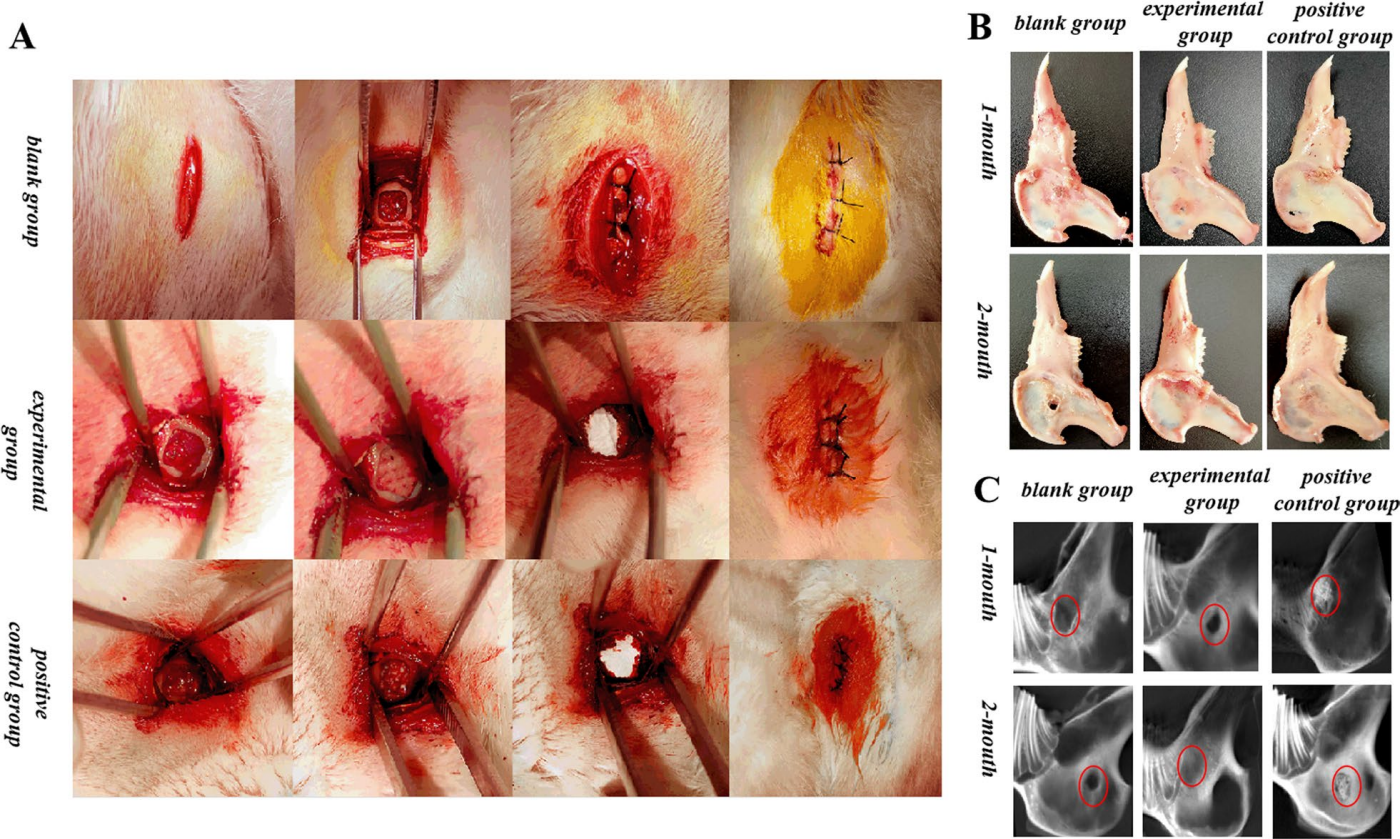
**A** Image of the surgical operation process; **B** Gross model of rabbit mandible; **C** Radiographic images

As can be seen from Fig. 6A–B, a small amount of bone deposits were visible in the blank space group during the first month after surgery. The Japanese white rabbits filled with Bio-Oss® particles were used as a positive group, it can be seen that there were new bone deposits in the bone defects, but undegraded bone particles were still visible. After implantation of 3D-printed scaffolds, the bone defects were not completely healed at the first month, but mandible obtained from rabbits at the second month almost healed by gross observing, showing an excellent therapeutic effect.

**Fig. 7:**
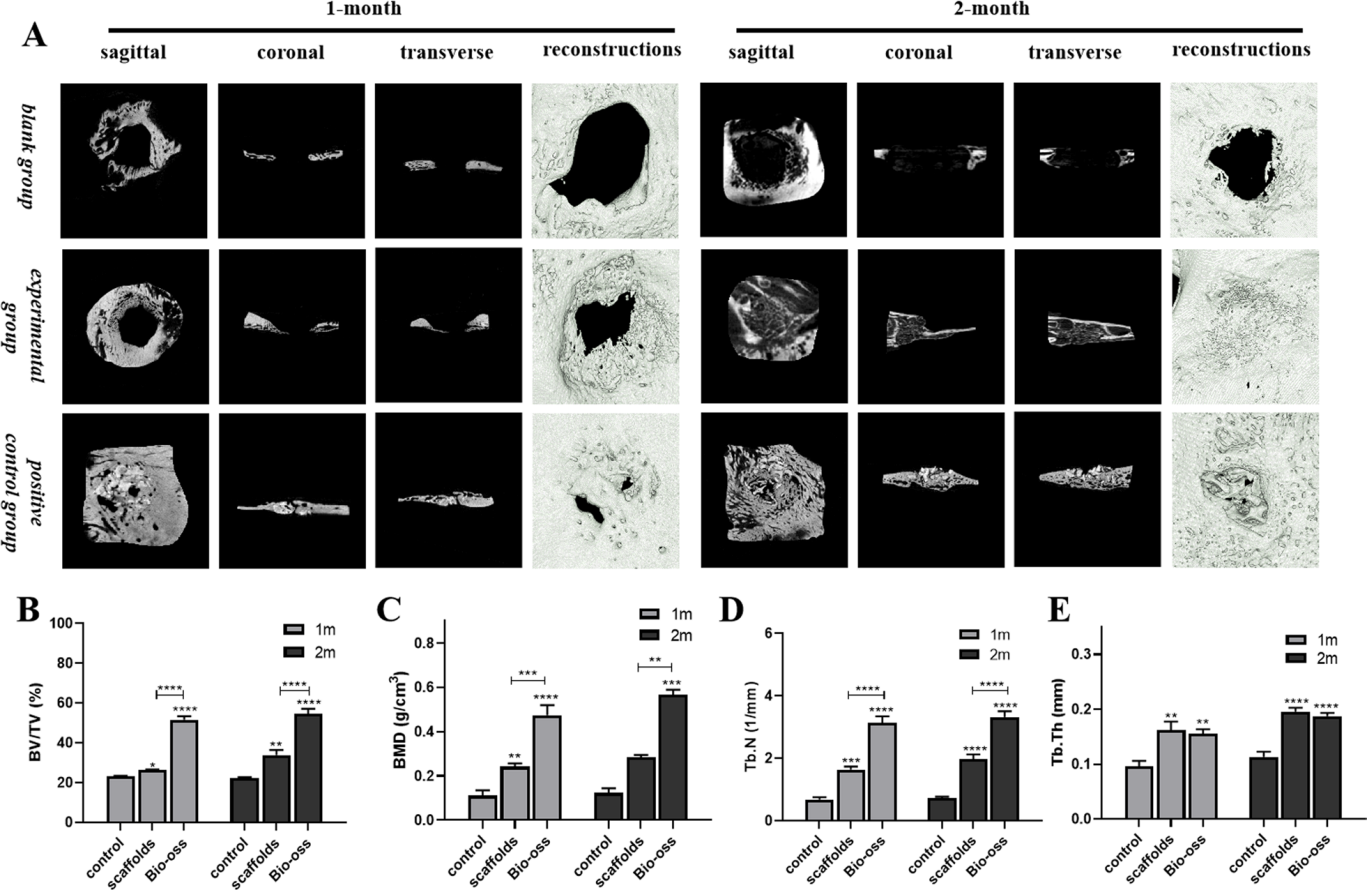
**A** Micro-CT scan results; **B**–**E** The statistical analysis graph of the bone volume fraction (BV/TV), bone mineral density (BMD), number of bone trabeculae (Tb.N) and trabecular bone thickness (Tb.Th), (“*”on behalf of *p* < 0.05, “**”on behalf of *p* < 0.01, “***”on behalf of *p* < 0.001, “****”on behalf of *p* < 0.0001)

From the analysis of CBCT (Fig. 6C) and Micro-CT (Fig. 7) analysis, it can be seen that the bone defects in the experimental group were significantly smaller and surrounded by newly formed bone at the first 1 month after the surgery, while obvious radiographic feature of Bio-Oss® particles was visible in the positive control group, indicating that the Bio-Oss® particles were not completely degraded at 1 month. Two months after surgery, the bone deposition was visible whether the positive control or experimental group, but the density of new bone tissue was significantly lower than that of the surrounding normal bone tissue on account of new bone formation, and although the density of the bone tissue in the positive control group was slightly higher, the Bio-Oss® particles were still not completely degraded.

Further morphometry analysis of the region of interest demonstrated that the BV/TV of groups 3D-printed scaffold were higher than that of black groups on the one of sacrifice (*p* < 0.05 in the first month and *p* < 0.01 in the second month), and the BMD of groups 3D-printed scaffold were also higher than that of black groups (*p* < 0.01). Meanwhile, the experimental group and positive control group also had significant more trabecular numbers than black groups (*p* < 0.01), and so did the trabecular thickness. (*p* < 0.01).

**Fig. 8:**
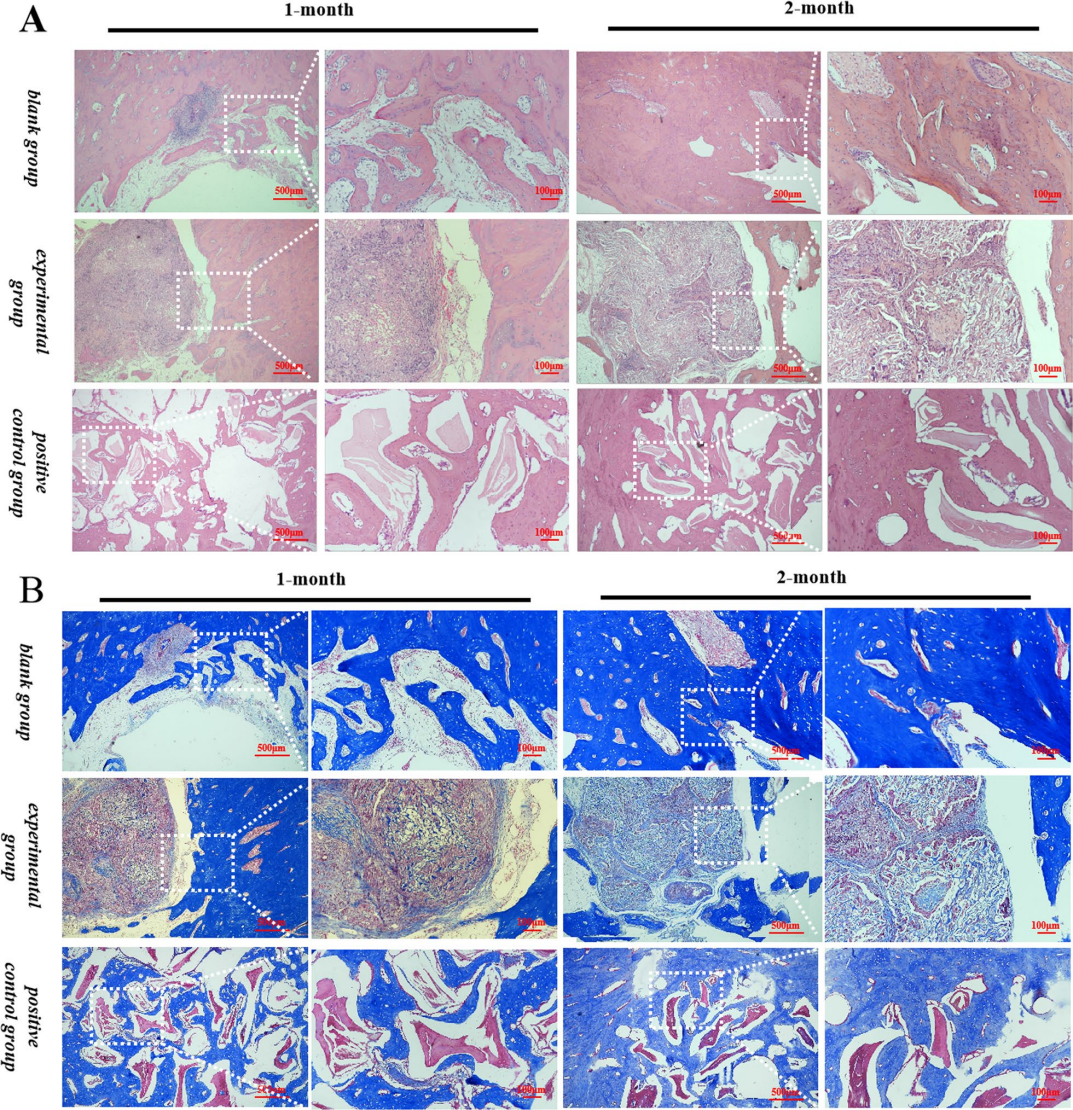
Results of HE and Masson staining in different periods

According to HE and Masson staining (Fig. 8), the osteoblasts could adhere and proliferate inside the scaffold, though the scaffolds were not completely degraded at two months, the regenerated osseous tissue could be seen inside the scaffolds, indicating that the scaffolds were gradually replaced by newly formed bone with the degradation of materials. Also, for the positive control group, a large amount of undegraded Bio-Oss® particles were still visible at month 1 and 2.

## Discussion

The bone defects caused by trauma, tumor or inflammation lesions are difficult to heal on their own, making a serious challenge for clinical work. Therefore, various bone graft materials are constantly being studied. The bone defects repair materials such as autogenous bone graft, allograft, xenograft and bone graft substitutes are most commonly used in clinic. Autologous cancellous bone contains a large number of osteoblasts, mesenchymal stem cells, growth factors and so on, meanwhile, cancellous bone matrix can act as a scaffold for the growth of osteoblasts and thus has osteogenic, osteoinductive and osteoconductive properties. While, there is a limited amount of autogenous bone available, and the use of autologous bone grafts may increase the risk of infection by opening up a second procedure site [31]. Allogeneic transplants, as well as heterologous graft, are expensive due to limited sources and complex production process, also may trigger immunological reactions and cause infection. Artificial bone grafts, such as calcium phosphate (CaP) or hydroxyapatite (HA) usually only possess osteoconductive properties, cannot meet clinical requirements when use alone. In recent years, the scaffold of bone tissue engineering is one of the research focuses currently. Jin et al. [32] evaluated the potential of the porous material composed of SF/HA as a scaffold for human placenta-derived mesenchymal stem cells in bone remodeling and showed that it could significantly promote the reconstruction and repair of bone. Ding et al. [33] designed a scaffold composed of HA and SF to load bone morphogenetic protein-2 (BMP-2), which improved the osteogenic ability and provided a good microenvironment for bone regeneration. Quinlan et al. [34] prepared a bioactive glass/collagen-glycoaminoglycan composite scaffold which significantly enhanced the production and expression of VEGF in endothelial cells, meanwhile, the composite scaffold had the ability to regulate the angiogenesis and proliferation of osteoblasts. However, most scaffold materials can only achieve osteogenic function, and cannot meet both of the antibacterial and osteogenic needs in the treatment of infectious bone defects. Therefore, this study is designed to develop a new-type scaffold material with antibacterial and osteogenic functions for repairing bone defects.

Apical periodontitis, an inflammatory disease with the destruction of periradicular tissues, is caused by specific microorganisms resulting in the persistence of inflammation and destruction of alveolar bone. Therefore, the ideal bone repair material should promote bone regeneration while inhibiting microbial growth. In general, patients are required to routinely take antibiotics for 3–5 days after endodontic surgery to prevent infection. Unfortunately, however, the abuse of antibiotics has posed a great challenge to modern medicine [35–37]. As a potentially effective medical treatment resource, antimicrobial peptides can rapidly destroy bacterial membranes and have difficultly making bacteria tolerant to the drug. However, since the discovery of antimicrobial peptides, they have not been widely used in clinical medicine due to their own shortcomings, such as unstable physicochemical properties, easy enzymatic hydrolysis and high cost, and their pleiotropic nature also limits their application as specific antibiotics [38]. However, the most important thing is that natural antimicrobial peptides need specific environments to be biologically active, suggesting that they have been largely influenced by host–pathogen interactions over millions of years of evolution [39]. Therefore, many natural antimicrobial peptides are sensitive to serum, divalent cations, pH value and protease, etc., which greatly limits their application range [40–42]. Whereas, synthetic antimicrobial peptides are expected to solve the above problems and become one of the hot areas of current research. KSL-W is a synthetic antibacterial peptide, which can effectively kill oral bacterial pathogens and inhibit oral biofilm formation. And the microsphere system prepared by PLGA can not only have an obviously sustained and controlled release effect on drugs, but also protect bioactive materials from the degradation of the corresponding environment and improve bioavailability, which has great potential for medical application [43–45].

In this study, KSL-W@PLGA microspheres were prepared by the W/O/W emulsifying-solvent evaporation technique. When microspheres loaded with water soluble drugs were prepared by the W/O/W emulsion method, the drug loading and encapsulation rate of microspheres were affected by many factors, including inner aqueous phase, the oily phase, the outer aqueous phase, polymer concentration, emulsifier, stirring speed, temperature, etc [18–22]. Therefore, the orthogonal test was designed to obtain the best preparation conditions to improve the encapsulation rate of KSL-W@PLGA microspheres. According to the results, we found that the concentration of PVA had the greatest impact on the encapsulation efficiency of the KSL-W@PLGA microspheres, followed by the concentration of the KSL-W and w/o ratio, while the concentration of PLGA was the least important factor. Therefore, the optimum technological conditions of preparation KSL-W@PLGA microspheres were determined, namely, the concentration of PLGA is 25 mg/mL, the concentration of KSL-W is 10 mg/mL, the concentration of PVA is 0.5%, and w/o ratio is 2/1.

KSL-W@PLGA microspheres were prepared according to the best technological conditions and the related properties were evaluated for subsequent experiments. Normally, the relationship between the concentration of KSL-W and absorbance value should first be determined when using the organic solvent dissolution method to calculate drug loading and encapsulation efficiency of KSL-W@PLGA microspheres. Therefore, we first drew the calibration curve of KSL-W, and it can be seen from Fig. 2B that the fitting coefficient is 0.99, suggesting the fitting effect was satisfactory. Also, it can be seen that when the concentration of KSL-W was between 0.1 and 1 mg/mL, the concentration values could be calculated based on the absorbance value. The drug loading and encapsulation efficiency of KSL-W@PLGA microspheres prepared in this experiment were (5.29 ± 0.05)% and (74.23 ± 0.24)%, respectively. In addition, SEM and CLSM were used to study surface morphology and drug distribution. Figure 2C, G indicated that the KSL-W@PLGA microspheres had a spherical structure with a smooth surface, and the uniform size was mainly concentrated at 10 μm. Figure 2D proved that the KSL-W was successfully loaded into the KSL-W@PLGA microspheres, however, a few shrunken microspheres could be observed, which might be caused by the powerful shearing force.

The evaluation of drug release behavior of KSL-W@PLGA microspheres was mainly characterized by measuring its content in the release solution (37 °C, 1 × PBS) at specific time points. From Fig. 2E–F, the rate of drug release of microspheres increases significantly in the first 5 days and later flattened, indicating that PLGA microspheres had a certain burst of release in the early stage. It could be inferred that the reason for this phenomenon may be that this stage was mainly the physical diffusion of drugs. The drugs attached to the surface of the microspheres could be quickly released into the PBS buffer solution, resulting in a faster drug release. After the sharp release period, the drug was encapsulated by PLGA and affected by the polymer chain, which hindered the diffusion of the drug and slowed down the release rate, tended to be stable.

Numerous studies have shown that *E. faecalis* and *P. gingivalis* are the main pathogenic bacteria of chronic periapical periodontitis. In most cases of endodontic treatment failure, one of the most common pathogenic microorganisms is *E. faecalis*, with a positive rate of 24–77% [46]. As a Gram-positive facultative anaerobe, it has the special ability to invade and colonize in the depths of the dentin tubules, withstand changes in extremes including calcium hydroxide (CH) and other drugs, and survive in a starvation environment. At the same time, the formation of *E. faecalis* biofilm in the root canal also increases the difficulty and failure rate of root canal treatment. In patients with periapical periodontitis secondary to RCT failure, 16S RNA genetic sequencing found that the number of *E. faecalis* was significantly increased [47]. *Porphyromonas gingivalis* also plays an important role in the occurrence and development of chronic periapical periodontitis, and its detection rate in the root canal samples of patients with periapical periodontitis was up to 69.4% [48]. Therefore, in this experiment, *E. faecalis* and *P. gingivalis* were used as experimental strains of bacteria to study the sustained antibacterial effect of KSL-W@PLGA microspheres. The experimental results showed that there was a significant bacteriostasis effect on the 5th and 10th days, indicating that the PLGA sustained-release microspheres prepared in the experiment could achieve sustained antibacterial effects. This provided a possibility for its application in bone defects with periapical inflammation.

In the process of studying bone substitutes to promote the healing of infected bone defects, we selected Bio-Oss®, an inorganic bone matrix derived from bovine material, which was commonly used in clinic, as the positive control group. The porous structure similar to the human alveolar bone, is conducive to the ingrowth of blood vessels and provides a scaffold for the ingrowth of osteoblasts [49]. However, it lacks osteogenesis and osteoinductive properties and is prone to collapse, also the absorption and degradation process in the body is very long [50]. Therefore, it is of great significance to develop new bone repair materials with good biocompatibility, suitable speed of biodegradation and antibacterial activity [51–53].

Collagen, as a natural organic component in animal skin, bone tissue, tendons, and ligaments, has good biocompatibility, biodegradability and low immunogenicity, and plays an important role in the adhesion, growth, migration and differentiation of cells. Collagen can promote new bone formation and is widely used in the regeneration of bones, blood vessels and nerves. However, its application in tissue engineering is limited due to the poor mechanical properties and the fast degradation rate [54]. Silk fibroin is a natural fiber, which has excellent performance in elasticity, flexibility, biocompatibility and biodegradability. In particular, the silk fibroin secreted by the silkworm has good biocompatibility and low immunogenicity, which has attracted widespread attention in the field of bone tissue engineering [55–57]. However, its low capacity of bone-forming limits its application in the realm of orthopaedics [58]. Hydroxyapatite (HA), as the main inorganic component of bone tissue, has good biocompatibility, stability and biodegradability [59]. It has little foreign body reaction and excellent osseous inductive ability. However, the main disadvantages of HA are brittle and not easy to be degraded. Therefore, researchers try to mix different biomaterials to achieve the best therapeutic effect. In this study, we prepared the KSL-W@PLGA/COL/SF/nHA scaffold and discussed its biocompatibility. The inoculation of MC3T3-E1 cells on the scaffolds was observed for 5-7d. HE staining and SEM were used to observe the growth of MC3T3-E1 cells. As can be seen from Figs. 4and5, MC3T3-E1 cells showed a tendency of multi-layer growth along the scaffolds aperture, and the cells adhered to the surface of the scaffold were fully stretched and the pseudopodia were visible. HE staining and SEM showed that the scaffold had good biocompatibility, which was conducive to the adhesion and proliferation of MC3T3-E1 cells.

The good three-dimensional structure and appropriate porosity play an important role in the adhesion and growth of cells, as well as for the exchange of nutrients and metabolites. Generally speaking, the mechanical properties of scaffolds will decrease when the porosity is too high; while the porosity is too low, it cannot meet the needs of osteoblasts to grow into the scaffolds and would affect the formation of new bone tissue. Zhang et al. [60] analyzed the bone tissue engineering scaffolds with 50%, 60% and 70% porosity from the macroscopic characteristics, microstructure and biomechanical properties, and found that the scaffolds with 60% porosity had the best effect. In general, the aperture sizes of scaffolds ranged from 150 to 800 μm [61–63]. Larger aperture size can increase the proliferation of osteoblasts and the growth of blood vessels. Meanwhile, sufficient nutrients exchange is also conducive to the formation of bone tissue, but mechanical properties are correspondingly reduced. However, when the aperture size of the scaffolds is too small, although there are certain advantages in mechanical properties, the limited adhesion and proliferation of cells and local hypoxia limit the bone repair ability. Generally speaking, the pore size suitable for bone tissue engineering is between 400 and 600 μm [64–67]. In our study, the porosity of the prepared 3D-printed scaffolds was (81.96 ± 1.83) % and the pore size was (523 ± 42) μm, which basically met the requirements of tissue engineering. Meanwhile, we studied the compressive properties of the KSL-W@PLGA/COL/SF/nHA scaffold with the elastic modulus being (57.93 ± 4.32) kPa. However, the range of elastic modulus for the cancellous bone is about 50–3000  MPa [68], so the insufficient mechanical strength of the scaffold may limit its clinical application.

To study the osteogenesis effects of KSL-W@PLGA/COL/SF/nHA scaffold, an infected bone defect model in the rabbit mandible was established. Considering that rabbits are rodents and their teeth grow continuously with chewing and attrition, it is not suitable to prepare the bone defect model of periapical periodontitis at the apex of the teeth. Therefore, the bone defects were prepared at the mandible of rabbits to simulate periapical periodontitis. In previous studies, New Zealand white rabbits were used to establish infected condyle defect model by creating a cylinder-shaped bony defect (6 mm × 4 mm) perpendicular to the femoral shaft to verify the HACC-grafted PLGA/HA scaffolds with significantly enhanced anti-infection and bone regeneration efficacy [30]. Li et al. reported a bifunctional M-CSH scaffold by building a round critical bone defect with a diameter of 6 mm at the mandibular body [28]. In this study, in order to adapt the bone defect model to the square 3D printed scaffold, we modified the construction method. Considering the small and thin bone plate of rabbit mandible, the circular bone defect with a diameter of 6 mm was modified to a square bone defect of 5 mm × 5 mm in order to ensure the chewing efficiency and survival rate of rabbits after surgery. The experimental groups of this study were 3D-printed scaffolds, which has the functions of inducing the bone formation and providing a three-dimensional environment in which cells can grow, while the Bio-Gide® membrane covered can prevent fibroblasts from growing into the bone defect area. Since Bio-Oss® particles and Bio-Gide® membrane have been widely used clinically as the ideal bone-repair materials, which have a significant clinical effect and favorable prognosis, they were used as the positive control group in this study. To verify the bone repair effect of KSL-W@PLGA/COL/SF/nHA scaffold, we performed HE and Masson staining (Fig. 8) of the operation site after 1-month and 2-month implantation. It was observed that there was a wealth of inflammatory cell infiltration in the black group compared with the scaffold group, the positive control group had the least inflammatory cells and only Bio-Oss® particles that had not degraded were visible. The osteoblast ingrowth was visible within the scaffold material at 1-month, indicating that the KSL-W@PLGA/COL/SF/nHA scaffold had good biocompatibility and could provide a scaffold for the proliferation of osteoblasts. On the day of sacrifice at 2-month, the KSL-W@PLGA/COL/SF/nHA scaffold in the bone defect region was not completely degraded, but the formation of bone tissue was visible, indicating that the scaffold material was gradually being replaced by newly formed bone tissue. We also performed radiographic analysis of bone healing status in rabbits. Micro-CT, as a micro-computed tomography technology, has a high resolution of tissue detection and does not damage the integrity of the sample, which has unique advantages in bone imaging. And at the same time, changes in bone tissue can be quantitatively analyzed by analyzing the parameters of each index in the target area. In this study, the values of BV/TV, BMD, Tb.N and Tb.Th in the experimental group were higher than those in the black group, especially in the 2-month. However, some parameters of positive control group, such as BV/TV, Tb.N, Tb.Th, etc., are abnormally increased, which may be attributed to the fact that Bio-Oss® particles itself are an inorganic bone matrix that removes all organic matter.

Broadly, the KSL-W@PLGA/COL/SF/nHA scaffold prepared in this experiment, has considerable cost benefits for its low cost and being rich of the raw material source. It has been demonstrated that the bone healing efficacy on mandible bone defects in rabbits, showing the potential for clinical application. However, this scaffold have some limitations due to insufficient mechanical strength. Still, there is no denying that, 3D printing technology has not been popularized, which increases the production cost for the preparation of scaffold materials.

## Conclusion

In this study, we successfully prepared the KSL-W@PLGA/COL/SF/nHA scaffold, which is a bone defect repair material with osteogenic activity and antibacterial function. The use of low-temperature deposition 3D printing technology can not only maintain the inherent activity of the material, but also prepare a three-dimensional porous scaffold that could adapt to the complex physiological environment and the diversity stress in the oral. We demonstrated that the scaffold possessed good biocompatibility and bone repairing ability. In summary, this 3D-printed scaffold may have potential applications in repairing infected bone defects.

## Supplementary Information

**Additional file 1.** **Table S1**: Factors and levels that influence the encapsulation rate. **Table S2**: The printing parameters. **Figure S1**: **A** A horizontal incision parallel to the lower margin of the mandible was made 2-3cm above the lower margin of the mandible **B** The exposed surgical field. **Figure S2**: Radiographic image of mandibular defect at month 0. **Table S3**: Absorbance values corresponding to different KSL-W concentrations. **Table S4**: The result of orthogonal test of KSL-W@PLGA microsphere.

## Data Availability

The datasets used and/or analysed during the current study are available from the corresponding author on reasonable request.
