# Supplementary material for: 3D printed scaffold for repairing bone defects in apical periodontitis
Source: BMC Oral Health. 2022 Aug 8;22:327. doi: 10.1186/s12903-022-02362-4 (PMC9358902; doi:10.1186/s12903-022-02362-4)
Supplement: Supplementary file 1 — Additional file 1. Table S1: Factors and levels that influence the encapsulation rate. Table S2: The printing parameters. Figure S1: A A horizontal incision parallel to the lower margin of the mandible was made 2-3cm above the lower margin of the mandible B The exposed surgical field. Figure S2: Radiographic image of mandibular defect at month 0. Table S3: Absorbance values corresponding to different KSL-W concentrations. Table S4: The result of orthogonal test of KSL-W@PLGA microsphere. [file 12903_2022_2362_MOESM1_ESM.docx]

**Electronic Supplementary Information**

**3D printed scaffold for repairing bone defects in apical periodontitis**

**Cong Li^1,^‡, Xiaoyin Xu^2,^‡, Jing Gao^1^, Xiaoyan Zhang^1^, Yao Chen^1^, Ruixin Li^1,^ *, Jing Shen^1,^ ***

**Experimental**

**Materials and characterization**

All chemical and biological supplies were purchased from Sigma-Aldrich. The peptide KSL-W was purchased from GL Biochem Co.,Ltd. (Shanghai, China), UV-vis absorption spectra were recorded on a Agilent Cary 60 spectrometer. Morphology study was performed by Scanning electron microscope (SEM, SU8100, HITACHI, Japan), laser particle scanning analyzer (MalvernZetasizerNanoZS90, Malvern Instruments Ltd., UK) and confocal laser scanning microscope (CLSM, Leica TCS SP8, Germany). The 3D scaffold model was designed using Solidworks 2010 (SolidWorks Corporation, Waltham, MA, USA), and printed with the 3D printer(OrganP 1800, Shangxian minimal invasive inc, China). Radiographic results were evaluated using 3DX550 Veraviewepocs (J. Morita Mfg Corp., Kyoto, Japan) and micro-CT(Skyscan, Germany). The Bio-Oss® particles and Bio-Gide® membrane were purchased from Geistlich Pharma AG (Wolhusen, Switzerland).

**The orthogonal test of the elements effecting KSL-W@PLGA microspheres**

The effects of the following four factors: (A) the concentration of PLGA, (B) the concentration of KSL-W, (C) the concentration of PVA emulsion and (D) w/o ratio on the encapsulation efficiency of KSL-W@PLGA microspheres were investigated. The experiment was adopted orthogonal design and chosen L_9_ (3^4^) orthogonal tables to repeat 3 times (Tab. S1).

**Fabrication of KSL-W@PLGA/COL/SF/nHA scaffold**

The 3D scaffold model was printed with the 3D printer. Set the printing parameters as shown in the Tab. S2.

**Construction of bone defect model**

The steps of making bone defect models were as follows:

(1) The Japanese white rabbits were injected intraperitoneally with 10% chloral hydrate (3.5mL/kg), and then the mandible of the rabbits were depilated with an electric shaver, disinfection the operation area with iodine followed deiodination with 75% ethanol.

(2) A 2-3cm horizontal incision was made at a distance of 2-3cm from the inferior margin of the rabbit's mandible. The scalpel touched the surface of the bone and cut the periosteum. The skin, connective tissue, muscle and periosteum were bluntly separated in sequence to expose the surgical field. Then, a 5mm×5mm square bone defect was made with a dental laboratory lathe.

(3) After 20 μL of the mixture of bacterial suspension of E. faecalis and P. gingivalis (1×10^6^ CFUs/mL) were placed in the bone defect for 5 minutes, they were sucked out with cotton swabs, and then implanted with different experimental materials, the muscle tissue and skin were sutured successively, and the surgical area was disinfected with iodophor. After the operation, all the Japanese white rabbits received the powder soft diet for one week. During the follow-up period, the bone infections in all groups were localized and no antibiotics were given after surgery.

**Tables**

Tab. S1 Factors and levels that influence the encapsulation rate

| Factors | | Experimental Parameters | | |
| --- | --- | --- | --- | --- |
|  |  | 1 | 2 | 3 |
| A | the concentration of PLGA（mg/mL） | 25 | 50 | 100 |
| B | the concentration of KSL-W（mg/mL） | 2.5 | 5 | 10 |
| C | the concentration of PVA emulsion（%） | 0.25 | 0.5 | 1 |
| D | w/o ratio | 2/1 | 1/1 | 1/2 |

Tab. S2 The printing parameters

| printing parameters | value |
| --- | --- |
| Given pressure (Pa) | 1 |
| General feed speed (mm/s) | 6 |
| Ordinary extrusion speed (mm/s) | 2.2 |
| first layer print height correction (mm) | 0.65 |
| print layer thickness(mm) | 0.42 |
| print layer | 7 |
| print needle diameter(nm) | 600 |
| bottom plate molding temperature(℃) | -20 |

Tab. S3 Absorbance values corresponding to different KSL-W concentrations

| Concentrations（mg/mL） | Absorbance values |
| --- | --- |
| 1.0 | 10.1983 |
| 0.8 | 8.1850 |
| 0.6 | 6.3976 |
| 0.5 | 5.6159 |
| 0.4 | 4.7457 |
| 0.2 | 2.6459 |
| 0.1 | 1.4763 |

Tab. S4 The result of orthogonal test of KSL-W@PLGA microsphere

|  | Factors | | | | encapsulation efficiency（%） |
| --- | --- | --- | --- | --- | --- |
|  | A | B | C | D |  |
| 1 | 1 | 1 | 1 | 1 | 35 |
| 2 | 1 | 2 | 2 | 2 | 72 |
| 3 | 1 | 3 | 3 | 3 | 41 |
| 4 | 2 | 1 | 2 | 3 | 44 |
| 5 | 2 | 2 | 3 | 1 | 39.5 |
| 6 | 2 | 3 | 1 | 2 | 38.4 |
| 7 | 3 | 1 | 3 | 2 | 25.6 |
| 8 | 3 | 2 | 1 | 3 | 22.6 |
| 9 | 3 | 3 | 2 | 1 | 68 |
| K1 | 148 | 104.6 | 96 | 142.5 |  |
| K2 | 121.9 | 134.1 | 184 | 136 |  |
| K3 | 116.2 | 147.4 | 106.1 | 107.6 |  |
| ‾K1 | 49.33 | 34.87 | 32 | 47.5 |  |
| ‾K2 | 40.63 | 44.7 | 61.33 | 45.33 |  |
| ‾K3 | 38.73 | 49.13 | 35.37 | 35.87 |  |
| R | 10.6 | 14.26 | 29.33 | 11.63 |  |
| The sequence of the factors | C>B>D>A | | | |  |
| Optimal Level | A_1_ | B_3_ | C_2_ | D_1_ |  |
| Optimal combination | A_1_B_3_C_2_D_1_ | | | |  |

**Figures**


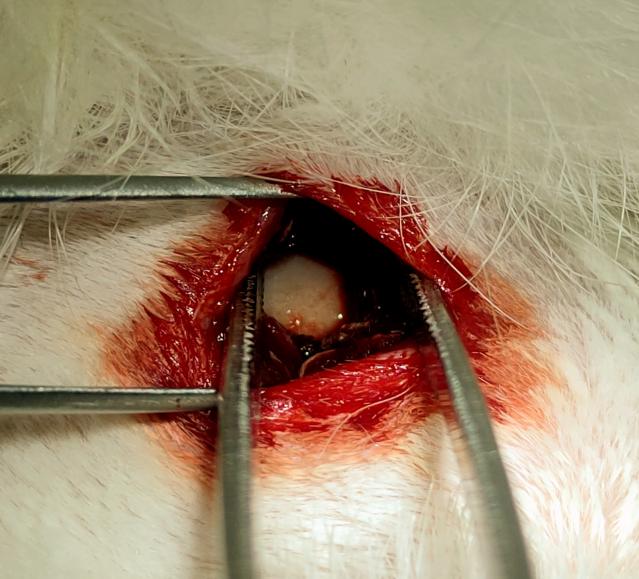

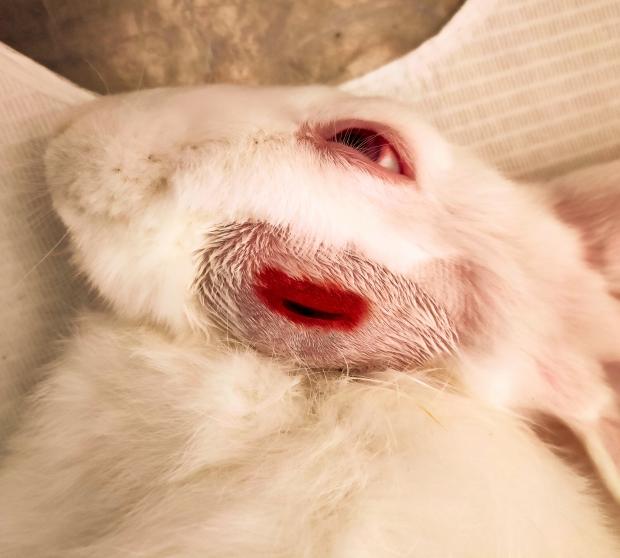


1. A
2. B

Fig. S1 (A) A horizontal incision parallel to the lower margin of the mandible was made 2-3cm above the lower margin of the mandible (B) The exposed surgical field


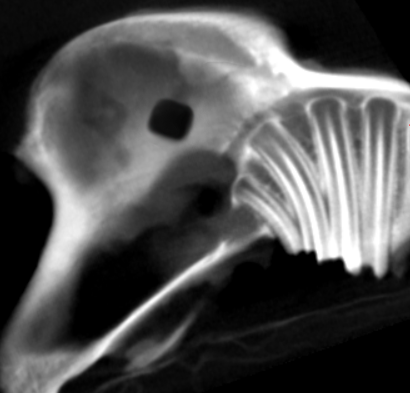


Fig. S2 Radiographic image of mandibular defect at month 0.
